# Mechanism of Inflammation in Age-Related Macular Degeneration: An Up-to-Date on Genetic Landmarks

**DOI:** 10.1155/2013/435607

**Published:** 2013-11-27

**Authors:** Francesco Parmeggiani, Francesco S. Sorrentino, Mario R. Romano, Ciro Costagliola, Francesco Semeraro, Carlo Incorvaia, Sergio D'Angelo, Paolo Perri, Katia De Nadai, Elia Bonomo Roversi, Paola Franceschelli, Adolfo Sebastiani, Michele Rubini

**Affiliations:** ^1^Department of Biomedical and Specialty Surgical Sciences, Ophthalmology Unit, University of Ferrara, Corso Giovecca 203, 44121 Ferrara, Italy; ^2^Department of Ophthalmology, Istituto Clinico Humanitas, Milan, Italy; ^3^Department of Health Sciences, University of Molise, Campobasso, Italy; ^4^Department of Ophthalmology, University of Brescia, Brescia, Italy; ^5^Center for Retinitis Pigmentosa of Veneto Region, ULSS 15 Alta Padovana, Camposampiero, Italy; ^6^Department of Biomedical and Specialty Surgical Sciences, Medical Genetic Unit, University of Ferrara, Ferrara, Italy

## Abstract

Age-related macular degeneration (AMD) is the most common cause of irreversible visual impairment among people over 50 years of age, accounting for up to 50% of all cases of legal blindness in Western countries. Although the aging represents the main determinant of AMD, it must be considered a multifaceted disease caused by interactions among environmental risk factors and genetic backgrounds. Mounting evidence and/or arguments document the crucial role of inflammation and immune-mediated processes in the pathogenesis of AMD. Proinflammatory effects secondary to chronic inflammation (e.g., alternative complement activation) and heterogeneous types of oxidative stress (e.g., impaired cholesterol homeostasis) can result in degenerative damages at the level of crucial macular structures, that is photoreceptors, retinal pigment epithelium, and Bruch's membrane. In the most recent years, the association of AMD with genes, directly or indirectly, involved in immunoinflammatory pathways is increasingly becoming an essential core for AMD knowledge. Starting from the key basic-research notions detectable at the root of AMD pathogenesis, the present up-to-date paper reviews the best-known and/or the most attractive genetic findings linked to the mechanisms of inflammation of this complex disease.

## 1. Introduction

Age-related macular degeneration (AMD) is the leading cause of irreversible central vision loss in elderly populations in developed countries, and 30–50 million people are affected worldwide. In the United States, it has been estimated that the prevalence of AMD is 13.4% among persons aged 60 years and older [1, 2]. AMD affects all retinal layers of the macula, the structure responsible for the central vision involving, in different degenerative patterns, photoreceptors, retinal pigment epithelium (RPE), and Bruch's membrane. AMD is primary characterized by the development of drusen, pathological extracellular deposits between RPE and Bruch's membrane mainly containing glycolipids, proteins, and cellular debris. At the level of macular area, the presence of few small hard drusen can be considered as a part of the normal tissue aging. On the other hand, AMD consists of numerous and large soft drusen, RPE dystrophy, macular pigmentary changes, and/or thickening of Bruch's membrane [3–8]. Advanced AMD can manifest as either dry form or wet one. In dry AMD, geographic atrophy with RPE and photoreceptors degenerative changes occurs. Conversely, wet AMD is characterized by the presence of choroidal neovascularization (CNV) with exudative and hemorrhagic phenomena leading to fibrotic scar formation [8–11].

Susceptibility to develop AMD is influenced by a number of genetic and environmental factors [12]. AMD is in fact considered a multifactorial disease, caused by the interplay between multiple acquired factors and polygenic background. Recent epidemiological data have identified numerous risk factors as potential modulators of AMD; aging, cigarette smoking, previous cataract surgery, and family history of AMD show strong associations with the disease, whereas higher body mass index, history of cardiovascular disease, hypertension, and higher plasma fibrinogen result in moderate AMD's risk [13]. Although family history is a well-known risk factor for AMD, it is not a routine practice to alert those with a family history to the increased risk and advise them of the relevance of recognizing early symptoms. An accurate quantification of risk associated with a family history of AMD has been reported in the UK population [14]. This study confirmed that family history is a strong risk element for AMD, highlighting the importance of genetic factors in the pathogenesis of AMD. Individuals with an affected sibling or an affected parent were reported to have a 12-fold increase in the odds of disease. Odds ratio (ORs) adjusted for age and smoking were even higher: 23% of siblings resulted to be affected by CNV or geographic atrophy with an OR of 10.8, which increased to 16.1 adjusting it for patient's age. Similar figure was previously reported in two large population-based studies, the Rotterdam Eye Study [15] and the Beaver Dam Eye Study [16]. The first reported an OR of 14.3, showing that the risk of AMD increased to 19.8 if smoking was taken into account. The latter study resulted in an OR of 10.3, which probably was underestimated, as the study relied on at least one sibling being free of disease at baseline and the sibships where all siblings were affected by AMD were excluded; when only randomly selected sibships were included, the OR was 26. Other studies reported family history of AMD being associated with lower ORs, but that could be attributed to the inclusion of cases with less severe disease [17–20]. A recent UK study has documented that the onset of disease was earlier in cases who had AMD affected siblings than in those without. This is typical for a complex disease with a substantial genetic component, for which the onset of disease is at a younger age in those with a family history [14].

Numerous genomic regions and a variety of candidate genes have also been seen to impact AMD susceptibility. Although strong associations between genetic factors and AMD have been illustrated, it is likely that a significant part of the heritability of AMD cannot be explained through current known mechanisms [21–23]. A number of genetic variants have been associated with AMD, and a recent genome-wide association study (GWAS) has provided significant (*P* < 5 × 10^−8^) evidence of nineteen AMD susceptibility loci [24]. Meta-analyses of GWASs for AMD estimated that currently identified loci account for approximately 60–70% of the inherited predisposition to clinically significant AMD forms [24–27]. The hugely mounting number of scientific reports regarding AMD-related gene variants counteracts the chance to get any unequivocal interpretation of these correlative data. In this complex scenario, which has built up more than ever in the last five years, several genes seem to be the most attractive in playing remarkable roles in different steps of AMD pathogenesis [24–37].

In the present review, we will focus on the best-known gene variants involved in the immunoinflammatory pathogenesis of AMD, particularly considering the regulation of both complement activity and cholesterol homeostasis.

## 2. Complement Activity

The complement system is one of the main components of the innate immune response and fulfils numerous functions, such as the recognition of foreign cells, communication with and activation of adaptive immunity, and the removal of cellular debris. Complement consists of over 40 proteins and cells, comprising a well-balanced network of circulating and cell-surface-bound proteins, which serve as substrates, enzymes, or modulators of a hierarchical series of extracellular proteolytic cascades. There are three well-known mechanisms of complement activation: classical, lectin, and alternative pathways. Each pathway is activated by different stimuli and the initial steps that trigger the complement activation differ considerably. The final stage of the enzymatic cascade of events is the lysis of bacteria or viruses and the opsonization, which consists in a sort of marking cells or molecules to be removed by the host [38–40].

The classical pathway is stimulated by the recognition of antigen-antibody complexes on foreign-cell surfaces by the hexameric complement component C1q. Similar pattern-recognition receptors, that is mannose-binding lectin (MBL) and ficolins, bind to carbohydrate ligands on microbial intruders and initiate the lectin pathway. Conversely, the alternative pathway is stimulated by the spontaneous hydrolysis of native C3 or the presence of foreign surface structures. Recent findings suggest that additional processes, such as the C2-bypass and extrinsic protease pathways or properdin-mediated direct convertase assembled on microbial surfaces, can also initiate complement activation [41–43].

All of the complement cascades end up in the central cleavage of C3 and in the generation of its active fragments C3a and C3b. Opsonization of foreign surfaces by covalently attached C3b fulfils three major functions:

- cell clearance by phagocytosis;
- amplification of complement activation by the formation of a surface-bound C3 convertase;
- assemblage of the C5 convertase.

Cleavage of C5 results in the formation of a multiprotein pore complex (MAC, membrane-attack complex), which leads to cell lysis. Both the covalent attachment of C3b and the stabilization of C3 convertase by the complement regulator properdin are markedly activated by hydroxyl-rich pathogen surfaces. A number of complement receptors mediate the recognition of opsonized cells by leukocytes, which induces phagocytosis and stimulation of the adaptive immune system (B and T cells). Finally, the anaphylatoxins C3a and C5a are released during complement activation and trigger a range of chemotactic and proinflammatory responses, such as recruitment of inflammatory cells and increase of microvasculature permeability. In this way, the complement cascade also supports and promotes the function of downstream mechanisms of the immune response [44, 45].

Detrimental effects take place in case of exaggerated complement activation on self tissue. In addition to a location-based and time-based restriction to immediate sites of activation, a finely tuned set of soluble and membrane-bound regulators ensure that any overstated action of complement on host cells is either prevented or actively inhibited. There is a large number of regulators of complement activation, including complement receptor 1 (CR1), complement factor H (CFH), factor H-like protein-1 (FHL-1), C4-binding protein (C4BP), decay-accelerating factor (DAF), and membrane cofactor protein (MCP) [46].

Complement is the most important pathogenic pathway of the immune system involved in AMD [8, 47–53], clearly indicating that complement activation is implicated in its pathogenesis [54–58]. Although AMD is not a classic inflammatory disease, immunocompetent cells, such as macrophages and lymphocytes, are present in chorioretinal tissues with AMD [59, 60]. Specific alteration and/or dysregulation of innate immune system are observed in AMD eyes mainly at the level of complement pathway elements, such as complement components C3a and C5a, C5 and C5b-9 terminal complement complex, complement regulators or inhibitors (i.e., CFH, vitronectin, and clusterin), CR1, MCP, and DAF, but also at the level of C-reactive protein [61–68]. Activation products C3a, C5a, and C5b-9 are also systemically elevated in patients suffering from AMD [69–72]. Due to genetic evidence from GWAS as well as from common and rare variant analyses, the overactive alternative pathway has been investigated showing that its excessive engagement is a key component in AMD pathogenesis [24–37, 54–58, 73–77]. During AMD, several immunopathological phenomena occur within the structures of the macular area, especially due to the pathophysiologic effects of complement system, which have a main role in the parainflammation of the aging retina [8, 47–53]. In particular, reliably because the posterior retinal layers (i.e., photoreceptor outer segment, RPE, and Bruch's membrane) are more prone to environmental and/or blood-circulating oxidative stresses [78–82], they epigenetically represent the preordained site of onset of the elementary AMD lesions (drusen) [3–8]. In fact, unregulated choroidal blood flow may increase the fluctuations of oxygen and/or lipids concentration, leading to elevated generation of reactive oxygen species (ROS) [80–82]. Likewise, photooxidation in photoreceptors is associated with complement activation, which can increase MAC formation, an important trigger of those apoptotic processes inducing retinal degeneration [83–86]. In this pathogenetic context, the critical position of complement must be emphasized. In fact, exactly complement's dysregulation can lead to that autologous damage which, at macular level, provokes the development of drusen: the earliest hallmarks of AMD acting as foci of chronic inflammation [8, 49–53].

## 3. Cholesterol Homeostasis

Recent investigation array has highlighted that neural retina and RPE express most of the genes involved in cholesterol homeostasis [87]. Indeed, it has been reported that retina can synthesize cholesterol endogenously [88, 89] and express proteins mediating cholesterol transport [90–92] and removal [93–95]. At the present time, detection of several cholesterol-related genes suggests that cholesterol homeostasis in the retina might be considered relatively independent of the rest of the human body. Taking part, respectively, in internal and external blood-retina barriers, endothelial cells of neural retina (NR) and RPE cells synthesize and acquire cholesterol from low- and high-density lipoproteins (LDL and HDL) derived from blood circulation. However, the ratio between blood-borne cholesterol and endogenously synthesized is not well-known yet [87, 96, 97].

A large interindividual variability of cholesterol and lipoprotein metabolisms is unquestionable, but it is intriguing the fact that RPE has higher variations in expression of cholesterol-related genes than NR. It could be accounted for a sort of “gate-keeping” function of RPE controlling cholesterol and nutrient uptake from blood-stream to NR and reverse transport of metabolites from NR back to systemic circulation. At RPE level this gene expression is promptly modulated in response to fluctuations of blood lipids. Moreover, this adjustment varies in each individual depending on blood lipid profile, age, gender, lifestyle, and genetic background. There are a lot of fine mechanisms of regulation pertaining to cholesterol-related genes in both NR and RPE but, despite the many experimental findings, most of them are not currently well known [87]. At NR level, it seems that photoreceptor outer segment (OS) deals with intraretinal cholesterol transport by means of active and passive mechanisms [98]. The active transport of cholesterol from photoreceptor inner segment (IS) to the OS partially occurs via intracellular cholesterol transporter Niemann-Pick C1-like 1 [99–101]. Another modality of cholesterol mobilization involves scavenger receptors, especially that named scavenger receptor Class B Member 1, which mediates bidirectional cholesterol flux between cells and lipoproteins; in this manner, the photoreceptor OS can uptake lipids from the HDL-like particles and offload lipids to the same particles as well [102–107]. Regarding the passive mechanism, it is known that photoreceptor IS lies in a high-cholesterol environment than OS; hence, IS can provide cholesterol for OS just through passive diffusion. Because of loss of efficiency in either some of these systems or phagocytosis, the cholesterol accumulates in the basal OS disks dampening down the phototransduction cascade [106, 108, 109].

In the retina, the RPE plays a key role in cholesterol homeostasis controlling both cholesterol input and output [110, 111]. Experimental findings have indicated the presence of different pathways, even if the cholesterol offload via apolipoprotein B-mediated transport is regarded as one of the main mechanisms involved in AMD pathogenesis. In fact, with aging, the apolipoprotein B-containing particles pool in the Bruch's membrane forming esterified and unesterified cholesterol-enriched lipid deposits named drusen. Very little is known about AMD and dysregulation of cholesterol-related genes, but it might be assumed that several affected individuals can be carrier of specific metabolic impairments in proteins determining cholesterol uptake (e.g., 3-hydroxy-3-methylglutaryl-CoA reductase and low-density lipoprotein receptor) and/or in those mediating cholesterol removal (e.g., ATP binding cassette transporter 1—*ABCA*1—cytochromes P*450*) [91, 92]. Although further investigation is needed to better elucidate these clinicogenetic relationships, recent GWAS identified four HDL-related genes as remarkable risk factors for AMD: *LIPC* (hepatic lipase), *CETP* (cholesteryl ester transfer protein), *ABCA1*, and *LPL* (lipoprotein lipase) [25, 31, 32].

In several age-related vascular disorders, increased levels of oxysterols play a crucial role provoking atherosclerosis with subsequent local and chronic inflammation. Homeostasis of cholesterol in blood vessel wall is of essential importance to regulate circulating cholesterol levels. A key event in the development of atherosclerosis is the uncontrolled uptake of oxidized LDL by macrophages recruited in the subendothelial space. The aberrant increasing of these lipid-loaded macrophages, termed foam cells, becomes a crucial condition causative of highly local inflammation [112–117].

Focusing on the lipoprotein retention in vascular wall, a parallel between atherosclerotic disease and AMD is identifiable. In atherosclerosis, apolipoproteins B cross the arterial endothelium, bind to proteoglycans, undergo oxidative and nonoxidative processes, and trigger downstream events, such as foam cells build-up and cytokine release [112, 118, 119]. In AMD, lipoprotein-like particles (enriched with esterified cholesterol) accumulate in the Bruch's membrane, especially in the space between the RPE basal lamina and the inner collagenous layer, forming lesions able to trigger inflammation, complement activation, and cytotoxicity (i.e., lipid-rich lesions, basal linear deposits and, finally, drusen) [3–8, 91, 92, 120–122]. RPE physiologically plays a critical role in the uptake, processing, and offload of retinal lipids. It uptakes the most part of oxidized lipoproteins via scavenger receptor Class B Member 3 and LDL receptors from the blood circulation, but it is also able to synthesize lipoproteins endogenously. On the other hand, aged or stressed RPE is unable to properly process the oxidized lipids, when the macrophages, which normally clean up these deposits, become less efficient and are slowly intoxicated by excessive levels of 7-ketocholesterol (7KCh) and other oxidized lipids [87–89, 96, 97, 123]. 7kCh is an oxidized form of cholesterol, that is, an oxysterol formed by auto-oxidation of cholesterol and cholesterol esters [124–127]; it is found in oxidized LDL deposits in the form of oxysteryl esters, covalently bound to oxidized unsaturated fatty acids [128–130]. Cholesteryl esters are particularly susceptible to oxidation and the cholesterol molecules in these esters can be oxidized to 7kCh [131–133]. In the primate retina, two main mechanisms for oxidation of cholesterol to 7kCh have been described: the Fenton reaction and the photooxidation [134–136]. The Fenton reaction requires a transition metal catalyst, such as iron and copper. Although the levels of these metals have not been measured in oxidized lipoprotein deposits, atherosclerotic plaques are known to contain relatively high levels of them [137]. By means of photooxidation and in presence of an adequate photosensitizer, cholesterol can be converted in a series of hydroperoxide intermediates that can further oxidize to 7kCh. Lipofuscin fluorophore A2E is one of the well-known photosensitizers, being involved in cholesterol ROS-mediated oxidation and also in the inhibition of the normal cholesterol efflux from RPE cells [138–140]. During the histopathologic evaluation of eyes affected by AMD, Lakkaraju and co-workers have documented that A2E induces aberrant cholesterol metabolism in RPE [140], which could contribute to AMD onset or progression also by means of inflammatory mechanisms.

## 4. Gene Variants Associated with AMD

Since 2005, several common variants in genes complement pathway have been consistently associated with the development of AMD. The common coding variant p.Tyr402His in the gene encoding complement factor H (*CFH*) was the first identified [64, 141–143]. A number of other polymorphisms in *CFH* [144], as well as in other genes involved in the alternative complement cascade, have also been demonstrated to affect AMD risk, including genes for complement component 2 (*C2*), complement component 3 (*C3*), and complement factor I (*CFI*) [29, 144–147]. More recently, common variants in genes encoding for cholesterol-related pathway, such as *LIPC* and tissue inhibitor of metalloproteinase 3 (*TIMP3*), have been reported to be associated with AMD in large GWASs [31, 32]. In the next subheadings, the genes implicated in phenotypic expression of AMD will be detailed, especially considering those main contributory variants at the basis of that immunoinflammatory dysregulation which, in AMD patients, can be labeled as inflammaging [148].

### 4.1. Complement Factor H

Originally known as *β*-1H globulin, CFH is a serum glycoprotein that regulates the function of the alternative complement pathway in fluid phase and on cellular surfaces. The binding of CFH to C3b reduces complement C3 activation, inhibits the formation of C3a, and lowers the production of IL-6 [149]. Besides, CFH accelerates the decay of the alternative pathway convertase C3bBb, and also acts as a cofactor for CFI, another C3b inhibitor [150, 151]. The *CFH* gene is located on chromosome 1q32, spans 94 kb, and comprises 23 exons. The *CFH* gene is located within a cluster of genes encoding the regulatory complement components of the activation of C3. This gene cluster includes the factor H-related genes *FHR1*, *FHR2*, *FHR3*, *FHR4*, and *FHR5* and the decay-accelerating factor, C4-binding protein (*C4BPA* and *C4BPB*), among others.

The c.1277 T-to-C transition in exon 9 of CFH gene (rs1061170) results in a substitution of histidine for tyrosine at codon 402 of the CFH protein (p.Tyr402His). This missense variant is located in the Short Consensus Repeat 7 (SCR7) that acts as a binding site of CFH to C-reactive protein (CRP) and heparin [152]. The binding of CFH to CRP or heparin increases CFH affinity for C3b and downregulates complement activity [153]. The p.Tyr402His can be considered to be a functional protein variant, as the p.His402 allele impairs the binding of CFH to CRP, thus resulting in an enhanced complement activation and consequent tissue damage. At sites of tissue injury, the p.His402 variant does not dampen the alternative pathway of complement activation as efficiently as p.Tyr402 allele [154–158]. In Caucasian populations of European ancestry the p.His402 allele is very common, having a gene frequency in the range of 0.3-0.4. The p.His402 allele is likely replacing the major one because in early life it provides a survival advantage against streptococcal infections; for example, microbes bind CFH to their surface to inhibit complement activation [46, 159]. The CFH binding protein of group A beta hemolytic streptococcus has a lower affinity for p.His402 than for p.Tyr402. As a result, the host's complement system has greater activity against the pathogen if the host expresses p.His402, thereby reducing the microbes' ability to counteract the alternative pathway. CFH adheres to damaged eukaryotic cells and tissue debris via the same anionic (heparin) binding sites that microorganisms employ to attach it to their surface [160–162]. If on one hand the p.His402 allele is potentially giving some benefit, on the other hand it is one of the most significant known genetic contributor to AMD disease risk. In individuals bearing a p.His402/His402 homozygous genotype, the risk of developing all categories of AMD was estimated to be 3-fold increased. Higher odds ratio (OR) values, in the range of 3.5–7.4, were found if only advanced dry and wet forms of AMD were considered [64, 141–143]. The association between the p.His402/His402 genotype and AMD could be explained by a reduced capacity of the p.His402 variant of CFH to bind debris in a damaged retina. Differential binding of p.His402 versus p.Tyr402 to multiple constituents of a damaged retina has been demonstrated for DNA, RNA, lipids, CRP, necrotic and apoptotic cells, heparin and other glycosaminoglycans, lipofuscin, bis-retinoids, photooxidation byproducts, and amyloid beta. The common finding is that the p.His402 protein binds with a lower affinity than p.Tyr402. Therefore, in the retina of a p.His402/His402 homozygous individual there is a higher level of alternative pathway activation, leading to retinal debris accumulation and ultimately AMD development.

Recent evidence has been reported supporting the existence of multiple AMD susceptible alleles in the chromosome region of the *CFH* gene [163]. A case-control study of 84 single nucleotide polymorphisms located in a 123 kb genomic region in 1q32 including the *CFH* gene provided evidence that multiple *CFH* haplotypes associate with AMD risk independently from p.Tyr402His [143]. Functional variants within these haplotypes are likely to influence the expression of *CFH* gene and possibly also of other nearby genes of the C3-activation cluster. In particular, an A-to-G variant located in intron 14 of *CFH* gene (rs1410996) has been reported to associate with AMD [144]. In the recent GWAS of Fritsche and co-workers [24], the most strongly AMD-associated single nucleotide polymorphism in the *CFH* region—rs10737680—was not in disequilibrium with p.Tyr402His, which instead was tagged by a weaker signal. This evidence further supports the hypothesis that multiple functional gene variants in the *CFH* locus act as risk factors for AMD.

### 4.2. *C2/CFB* Cluster

The *C2* gene encodes for the complement component 2, spans 18 kb, and includes 18 exons. The *C2* gene maps in 6p21.33 and is adjacent to the *CFB* gene, which encodes for complement factor B, from which is separated by just 271 nucleotides. Common variants within the *C2/CFB* cluster have been recently confirmed as being significantly associated with risk of developing AMD [164].

A missense G-to-C variant in exon 7 of C2 (rs9332739) has a frequency of 0.067 among Europeans and causes the replacement of the glutamic acid residue at codon 318 with an aspartic acid one (p.Glu318Asp). The C-to-A substitution within intron 10 of C2 (rs547154) has a frequency of 0.062 among Europeans. Minor alleles of both rs9332739 and rs547154 have a protective effect and reduce by half the risk for developing AMD. A recent meta-analysis estimated that OR of C-allele of rs9332739 was 0.55 (95% confidence interval (CI): 0.46, 0.65), while minor allele at rs547154 carried an OR of 0.47 (95% CI: 0.39, 0.57) [27].

The CFB:c.26 T-to-A transversion (rs4151667) in exon 1 of *CFB* results in the substitution at codon 9 of leucine with histidine (p.Leu9His) that has a frequency of 0.067 among Europeans. A second missense variant in *CFB* gene, the CFB:c.95 G-to-A transition (rs641153), is located in exon 2 and determines a substitution of arginine at position 32 with a glutamine residue (p.Arg32Gln). The minor A-allele of CFB:c.26 T-to-A and the A-allele of CFB:c.95 G-to-A carried estimated risks of 0.54 (95% CI:0.45, 0.64) and 0.41 (95% CI:0.34, 0.51), respectively [27].

Haplotype analyses using two independent cohorts of AMD patients identified a statistically significant common risk haplotype and two protective haplotypes [145]. Both the haplotype including minor alleles of C2:c.954 G-to-C and CFB:c.26 T-to-A (H10 haplotype) and the haplotype including A-allele of rs547154 variant in intron 10 of C2 and the A-allele of CFB:c.95 G-to-A (H7 haplotype) confer a significantly reduced risk for AMD [145]. The protective effect of H7 haplotype has been confirmed in independent studies, and evidence has been reported suggesting that minor alleles of both variants contribute independently to the protective effect. To date it is not clear if the rs547154 variant in intron 10 of C2 has a functional activity or rather is in disequilibrium with a causal variant, but it is likely that its minor allele could be associated with a lower expression of C2. The CFB protein containing glutamine at position 32 has been reported to reduce hemolytic activity compared with the arginine containing form and to cause less efficient complement activation [165, 166]. This lower complement response determined by H7 haplotype could possibly explain the protective effect on AMD development. Combined analyses of the *C2/CFB* haplotypes and *CFH* variants showed that variation in the two loci can predict the clinical outcome in 74% of the affected individuals and 56% of the controls [167].

### 4.3. Complement Component 3

The *C3* gene encodes the complement component 3, a factor that plays important biological roles in the classical, alternative, and lectin activation pathways. The *C3* gene spans 41 kb on chromosome 9p13.3 and comprises 41 exons. The active C3 factor includes an *α*-chain, encoded by the last 26 exons, and a *β*-chain, encoded by the first 16 exons, having exon 16 encoding both *α* and *β*-chain. The synthesis of C3 factor is induced during acute inflammation. C3 is produced mainly by liver but also by activated monocytes and macrophages. Mature C3 factor is obtained from the cleavage of a single chain 200 kDa precursor into the *α* (C3*α*) and *β* (C3*β*) subunits that are linked by disulfide bonds. C3 factor has a critical role in the complement system, and C3 deficiency makes people more susceptible to bacterial infection. The c.304 C-to-G substitution in exon 3 of *C3* gene (rs2230199) is a common missense variant that causes replacement of arginine residue at codon 102 with a glycine one (p.Arg102Gly). These two alleles correspond to the slow and fast electrophoretic variants of C3 factor. The p.Gly102 allele has a frequency of 0.175 among Europeans and is carried by more than 30% of individuals. Association between p.Arg102Gly and AMD has been confirmed in many studies on Caucasian populations [55, 146, 147, 164, 168, 169], but not in Asian populations, probably due to the lower frequency of the p.Gly102 allele [170]. Among Europeans, the OR for AMD has been reported to be 1.7 in p.Arg102/Gly102 heterozygotes and 2.6 in p.Gly102/Gly102 homozygotes, and the estimated population attributable risk for p.Gly102 was 22% [147].

### 4.4. Complement Factor I

The complement factor I (CFI) gene maps on chromosome 4q25, spans 63 kb, and comprises 13 exons. The first eight exons encode the heavy chain of CFI, while the light chain of CFI is encoded by the last five exons. The two chains are linked by disulfide bonds. CFI is a serine protease that plays a role in the complement pathway as it cleaves and inactivates C4b and C3b. A C-to-T transition (rs10033900) located 4.3 kb downstream the 3′ UTR of *CFI* gene has been shown to be independently associated with AMD [29, 31]. This variant could have a role in influencing CFI expression level or be in linkage disequilibrium with a functional regulatory variant.

### 4.5. *ARMS2/HTRA1* Locus

A locus in 10q26.13 (LOC387715) has been identified as the second most important locus in the etiology of AMD [170, 171]. This locus includes the age-related maculopathy susceptibility (ARMS2) gene and the gene encoding for the high-temperature requirement factor A of serine peptidase 1 (HTRA1). A G-to-T transversion in exon 1 of ARMS2 (rs10490924) is a common missense variant that replaces an alanine residue with a serine (p.Ala69Ser). The p.Ser69/Ser69 homozygotes were reported to have a significant 7.6-fold increased risk of developing AMD [171], and this association has been next confirmed in independent case-control studies. Only 4.2 kb separate *ARMS2* gene from the near *HTRA1* gene, and the ARMS2:p.Ala69Ser variant is located just 6.3 kb from a G-to-A variant in the promoter region of HTRA1 gene (rs11200638). These two variants are in strong linkage disequilibrium (*r* ^2^ = 0.90), and it is difficult to determine which one could be the causal variant in this locus [24]. Therefore, it is still under debate to definitively establish which gene, *ARMS2*, *HTRA1* or possibly also others, is responsible for the genetic association with AMD [8].

The *HTRA1* gene encodes a member of the trypsin family of serine proteases. The HTRA1 protein is a 50 kDa secreted enzyme that cleaves substrates involved in the complement pathway, such as clusterin, vitronectin and fibromodulin, and could theoretically play a role in the pathogenesis of AMD. The G-to-A substitution in the promoter region of HTRA1 has been initially considered a functional variant as it is located in a conserved CpG island and resides within a putative binding site for the transcription factor adaptor-related protein complex-2*α* and could possibly regulate the expression level of HTRA1 [172, 173]. However, later studies showed that this variant does not affect the transcription level of HTRA1 in several cell lines [174] nor alters HTRA1 mRNA or protein expression in human retina-RPE-choroid samples [175]. Therefore, it is unlikely that rs11200638 is the functional variant that accounts for the strong association between the *ARMS2*/*HTRA1* locus and the risk of developing AMD.

*ARMS2* is a small gene—just 2.7 kb wide—that includes only two exons and a single intron. The encoded 107-amino acid peptide is expressed in the outer membrane of mitochondria and in the citosol. The p.Ala69Ser variant could affect the conformation of protein and eventually modify mitochondria function [174]. A second variant, an insertion/deletion (indel) polymorphism in the 3-prime untranslated region (3′UTR) of *ARMS2* (ARMS2:c.372_815del443ins54), has been strongly associated with risk of developing AMD (*P* = 4.1 × 10^−9^) [164]. The association between del443ins54 indel and AMD has been replicated in different populations [176]. This indel variant removes the polyadenylation signal in the 3′ UTR of ARMS2 and replaces it with a 54 bp element known to mediate rapid mRNA turnover. The expression of ARMS2 transcript is lost in homozygous carriers of the del443ins54 indel. This variant is located between ARMS2:c.269 G-to-T and HTRA1:c.-625 A-to-G, and the haplotype including minor alleles (T-indel-A) was reported to be associated with a significant 3-fold increased risk for AMD [176]. Considering the deleterious effect of del443ins54 indel to the expression of *ARMS2* transcript, we could suggest that this indel polymorphism could be the actual variant causing the increased risk of AMD associated with the *ARMS2/HTRA1* locus.

The actual function of ARMS2 protein is unknown, but it is thought to play a role in diseases in the elderly [8]. *ARMS2* transcripts have been detected in retina and in a variety of other tissues and cell lines [174], and it has been proposed that ARMS2 could play a key role in AMD through mitochondrial-related pathways [164]. So far, very little is known about the function of ARMS2, and more investigations are needed to determine if variants in this gene have causal role in the pathogenesis of AMD.

### 4.6. Tissue Inhibitor of Metalloproteinase 3

The tissue inhibitor of metalloproteinase 3 (*TIMP3*) gene belongs to a family of genes encoding for inhibitors of matrix metalloproteinases, a group of zinc-binding endopeptidases involved in the degradation of the extracellular matrix. TIMP3 is also a potent angiogenesis inhibitor, as it blocks the binding of VEGF to VEGFR2 and inhibits downstream signaling leading to VEGF-mediated angiogenesis [177]. The *TIMP3* gene spans 55 kb on chromosome 22q12.3 and includes 5 exons. *TIMP3* mutations are causing a Mendelian early onset form of macular degeneration often complicated by CNV, known as Sorsby's fundus dystrophy (MIM #136900). *TIMP3* has been considered a putative candidate for AMD susceptibility, but early studies failed to find association between TIMP3 and AMD [178, 179]. Recent evidence has been reported indicating that an A-to-C substitution located far upstream (113 kb) of TIMP3 gene (rs9621532) within an intron of the synapsin III gene (SYN3) is associated with a reduced risk of developing AMD [31]. This variant influences the expression of TIMP3 transcripts in cultured primary human fetal RPE cells, and the protective C-allele of rs9621532 was associated with mRNA expression [180]. However, the genetic association between rs9621532 and AMD has not been confirmed in Asian population, and the role of TIMP3 in AMD etiology still remains controversial [181].

### 4.7. Hepatic Lipase

*LIPC*, a novel AMD gene, is involved in HDL cholesterol metabolism. The gene spans 60 kb on chromosome 15q21.3, includes 9 exons, and encodes a hepatic triglyceride lipase which is expressed in liver. LIPC enzyme is also a triglyceride hydrolase and a ligand/bridging factor for receptor-mediated lipoprotein uptake. Rare deficiencies of LIPC are associated with pathologic levels of circulating lipoprotein. Expression of LIPC in the retina has been reported [167]. Two variants in *LIPC* putative promoter, an A-to-G substitution (rs493258) located 14 kb from LIPC transcription start site and a C-to-T substitution (rs10468017) 22 kb upstream of *LIPC*, were reported to be associated with advanced AMD in two independent European cohorts, indicating that common variants in LIPC gene could play a role as genetic risk factor for AMD [31, 32, 182]. These variants are thought to regulate the expression of *LIPC* and therefore influence the metabolism of HDL cholesterol. The T-allele of rs10468017 has been reported to have a protective effect for advanced wet and dry AMD by influencing LIPC expression in serum and increasing HDL levels [32].

## 5. Final Remarks

This etiogenotypic excursus has been first and foremost aimed to speculatively interconnect two different types of gene polymorphism, which are able to alter either complement or cholesterol pathway and, consequently, to predispose to AMD via inflammation and parainflammation. Several clinicogenetic studies show increased OR to develop AMD in individuals carrying more risk genotypes [167, 183]. In particular, the carriers of combination of peculiar *CFH*, *ARMS2/HRTA1*, and *C2/CFB* genotypes have been reported to have high OR values, although significance level of these findings were relatively low, mainly due to the low number of patients included in the investigations. Calculating a risk score including genetic information across the nineteen top loci resulting from a very recent GWAS [24], Fritsche and co-workers reported that a multiple combination of genotypes could distinguish AMD patients from healthy controls (area under the receiver operator curve = 0.74) and suggested that similar scores could be used to identify and prioritize at-risk individuals, in order to provide them preventive treatment before the disease onset. The development of an efficient tool able to predict the development of AMD is strongly awaited, as it could have a remarkable impact on the health systems. However, several biases can counteract the expectation to achieve reliable data on this complex topic. Many small clinicogenetic studies and, despite adequate statistical protection from multiple comparisons, some GWASs are at risk of findings by chance or of overestimating marker effects [184, 185]. Therefore, correct translational information from genomic marker research to clinical practice of AMD will be more rapidly available if biomedical community works together in carrying out large-scale consortium of trials designed to concomitantly verify the weight of both clinical [13] and genotypic [24] risk factors in AMD patients, as recently performed by Seddon and co-workers in a quite numerous sample population [186]. Before long, the validation of risk prediction models, inclusive of proteomic biomarkers, will be useful for the managing of research, clinical trials, and personalized medicine not only in AMD, but also in other frequent causes of legal blindness such as diabetic retinopathy, glaucoma, and pathologic myopia [1]. In particular, AMD risk scores based only on simple sums of genotypes are unlikely to turn out effective, probably because the complex nature of AMD etiology includes synergistic interactions both among gene variants and among these and environmental conditions. A more comprehensive exploratory approach on the relationship between the chief AMD-risk genotypes, the underlying immunoinflammatory endophenotypes, and the networks of interaction with acquired or epigenetic factors is likely to provide, in the near future, the knowledge for the development of useful predictive algorithms, able to guide in the direction of an effective primary and secondary prevention of AMD.
